# Economic Effect of Smoke-Free Ordinances on 11 Missouri Cities

**DOI:** 10.5888/pcd9.110277

**Published:** 2012-05-31

**Authors:** Noaman Kayani, Stanley R. Cowan, Sherri G. Homan, Janet Wilson, Victoria Fehrmann Warren, Shumei Yun

**Affiliations:** Author Affiliations: Noaman Kayani, Sherri G. Homan, Janet Wilson, Victoria Fehrmann Warren, Missouri Department of Health and Senior Services, Jefferson City, Missouri; Stanley R. Cowan, University of Missouri–Columbia School of Medicine, Columbia, Missouri.

## Abstract

**Introduction:**

The harmful effects of secondhand smoke are convincing more and more communities across the United States and the world to prohibit smoking in public places, especially in eating and drinking establishments. A 1993 Missouri state law allows smoking in designated areas in indoor public places such as restaurants and bars. Consequently, some Missouri communities have adopted local ordinances that prohibit smoking in all indoor workplaces, including restaurants and bars. We used an objective measure of economic activity, the taxable sales revenues of eating and drinking establishments, to empirically examine the economic effect of smoke-free ordinances.

**Methods:**

We studied the economic effect of smoke-free ordinances in 11 Missouri cities using multivariate log-linear regression models with log-transformed taxable sales revenues of eating and drinking establishments as the dependent variable and the smoke-free ordinance as the independent variable, while controlling for seasonality, economic condition and unemployment. We used data from 20 quarters before the smoke-free ordinances and at least 10 quarters after the smoke-free ordinances for all cities. The null hypothesis of no effect of smoke-free ordinance on taxable sales of the eating and drinking establishments was tested.

**Results:**

Eight of the 11 cities had increased taxable sales for eating and drinking establishments postordinance. The remaining 3 experienced no change.

**Conclusion:**

The findings of our study are consistent with findings from most published economic studies that a smoke-free ordinance does not harm a local economy.

## Introduction

Tobacco use is responsible for approximately 1 in 5 deaths in the United States, or 443,000 deaths per year; exposure to secondhand smoke is estimated to cause 49,000 of these tobacco-related deaths (1). The economic cost of cigarette smoking is more than $193 billion, including $10 billion that results from secondhand smoke costs in terms of health care expenditures, illness, and death (1,2).

Adopting smoke-free policies can be a wise health and business decision. However, when a local government or legislature considers smoke-free ordinances or laws, the issue of economic impact is usually raised. Opponents claim that the ordinance will negatively affect the business of local restaurants and bars. A sizable body of evidence continues to accumulate on the economic effect of smoke-free legislation as it relates to restaurants, bars, and other hospitality venues.

A review of published economic studies found that smoke-free ordinances did not have an adverse economic effect in diverse localities such as New York City; Boston; Minneapolis/St. Paul; Lexington, Kentucky; or several cities in Texas (3-6). In Missouri, 3 studies examined the economic impact of smoke-free ordinances adopted in Maryville, Columbia, and Kansas City (7-9). The Maryville study found that the ordinance was associated with increased revenue, and the other 2 studies did not find any effect. None of these studies were published in a peer-reviewed journal.

The objective of this study was to examine whether smoke-free ordinances have any effect on sales of eating and drinking establishments using an objective measure of taxable sales revenues in 11 cities in Missouri.

## Methods

Consistent with earlier studies (10-13), we conducted a multiple log-linear regression analysis, separately for each city, using SAS version 9.2 (SAS Institute, Inc, Cary, North Carolina). Cities included Ballwin, Blue Springs, Chillicothe, Columbia, Independence, Kansas City, Kirksville, Lee's Summit, Maryville, Nixa, and North Kansas City, all of which had at least 10 quarters of taxable sales data available postordinance as of and including December 2010 (Box). We did not include communities that had a smoke-free ordinance but did not have sufficient quarters of postordinance data to analyze the effect. These included Brentwood, Clayton, Creve Coeur, Fulton, Jefferson City, Kirkwood, Lake St. Louis, Liberty, St. Louis City, O'Fallon, St. Louis County, Springfield, and Warrensburg.

**Box. Smoke-Free Ordinances in Missouri Cities With Effective Dates Before 2011**

**Table:** 

**City**	**Smoke-Free Ordinance Effective Date**	**Type of Ordinance**
Ballwin	March 11, 2005	All workplaces, including eating and drinking establishments
Blue Springs	May 1, 2008	Allowed in non–publicly accessible areas of workplaces and public places, eg, employee break rooms
Chillicothe	June 1, 2008	All workplaces, including eating and drinking establishments
Columbia	January 7, 2007	All workplaces, including eating and drinking establishments
Independence	March 17, 2007	All workplaces, including eating and drinking establishments
Kansas City	June 23, 2008	All workplaces, including eating and drinking establishments, but exempts casino gaming floors
Kirksville	July 1, 2007	Restaurants and bars
Lee's Summit	March 17, 2007	All workplaces, including eating and drinking establishments
Maryville	July 1, 2003	Restaurants^a^
Nixa	June 8, 2007	Allowed in nonpublicly accessible areas of workplaces and public places, eg, employee break rooms
North Kansas City	August 11, 2008	All workplaces, including eating and drinking establishments, but exempts casino gaming floors
^a^ In 2010, Maryville expanded its ordinance to include bars; however, in the analysis we used the initial ordinance date of July 1, 2003.		

### Data sources

We used quarterly taxable sales data from the Missouri Department of Revenue, which reports total revenues and revenues for eating and drinking establishments under the standard industrial classification code 581 (14).

For this analysis, preordinance data included 20 quarters of data covering 5 years before each ordinance's enactment, and postordinance data included all quarters after the ordinance's effective date through December 2010, which ended the most recent quarter of data available.

To adjust for inflation we used the consumer price indices (current series) for all urban consumers for the Midwest region published by the Bureau of Labor Statistics (15). Unemployment data were obtained from the Missouri Economic Research and Information Center for macropolitan and micropolitan statistical areas. When the city's unemployment data were not available, we used the county unemployment rate (16).

### Data analysis

In the log linear regression analysis, the dependent variable *Ln EDRev*, the natural log of inflation-adjusted taxable sales revenues of eating and drinking establishments (EDRev), was regressed on several explanatory variables as follows: *Ln* *EDRev* = β_0_ + β_1_ *Quarter*2 + β_2_ *Quarte*r3 + β_3_ *Quarter*4 + β_4_ *Ordinance* + β_5_ *Ln Economic Activity* + β_6_ *Unemployment* + ε

The EDRev were log-transformed to achieve homogeneity in the error structure. To capture seasonal fluctuations, 3 quarterly dummy variables were added. To see if the smoke-free ordinance significantly changed the EDRev, we included a dichotomous variable, *Ordinance*, that takes the value of "0" for preordinance and "1" for postordinance data. To control for the city's economic conditions and the demand for eating and drinking establishments, *Ln Economic Activity*, the natural log of the city's inflation-adjusted total taxable sales (TOT) net of revenues from the eating and drinking establishments (TOT – EDRev) and unemployment rates were used in the analysis.

Initially, we included a time trend variable and its interaction term with *Ordinance*. We detected a multicollinearity problem on the basis of a variance inflation factor greater than 10. When the trend variable and its interaction with the ordinance variable were excluded from the model, the above reduced model did not have an issue with multicollinearity except with the city of Independence. For Independence, we additionally excluded *Ln Economic Activity* from the model.

We performed other model diagnostic procedures to check for linearity, normality, equal variance, independence of variance, and outliers. Using a Cook's D of 1 or more as a criterion, we detected no outliers, and we conducted the Durbin-Watson test for first-order serial correlation. We used the critical values of Durbin-Watson when quarterly dummy and/or trend variables were included as the regressors (17). The first-order serial correlation was detected in all of the cities except Columbia and Kansas City, so we used the Newey-West estimation technique for correction of standard errors.

In the case of significant change after the smoke-free ordinance, we computed both percentage change and the dollar amount of change in taxable sales revenues. The percentage change between preordinance and postordinance was estimated by exp (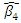) – 1. The amount of change was computed as *EDRev_post_ – EDRev_pre_*, estimated using a value of "1" for post and "0" for pre, for the *Ordinance* variable, and the average values for other explanatory variables in the model.

The null hypothesis of no effect of smoke-free ordinance on taxable sales of the eating and drinking establishments was tested using 2-tailed *t* tests.

## Results

Overall, the smoke-free ordinance was associated with a significant increase in revenue for eating and drinking establishments in 8 of the 11 cities (Table). For the other 3 cities, we were unable to detect a significant effect of the ordinance on the taxable sales revenues. The largest relative increase in revenue was in Nixa (36.5%), followed by Maryville (18.6%) and Lee's Summit (10.4%). No significant change was identified in Ballwin, Kansas City, or Chillicothe. Ballwin is in the suburb of St. Louis County in eastern Missouri; Kansas City is a large city on the western border; Chillicothe, in northern Missouri, is the most rural city in the analysis. There is limited or no commonality among the 3 cities.

**Table:** Effect of Smoke-Free Ordinances (SFOs) on the Revenues of Eating and Drinking Establishments in 11 Missouri Cities^a^

City	No. of Quarters of Data Used		Factors Controlled for^b^	ƈ for SFO	*P* Value	Change in Revenue, $ (%)^c^

	Pre-SFO	Post-SFO				
Ballwin	20	23	All	–0.038 (0.043)	.39	NC
Blue Springs	20	11	All	0.058 (0.011)	<.001	844,339 (5.9)
Chillicothe	20	10	All	–0.027 (0.032)	.41	NC
Columbia	20	16	All	0.045 (0.015)	.11	2,059,643 (4.6)
Independence	20	15	All except economic condition	0.037 (0.010)	.001	1,452,206 (3.8)
Kansas City	20	10	All	0.033 (0.040)	.42	NC
Kirksville	20	14	All	0.096 (0.037)	.01	484,159 (10.1)
Lee's Summit	20	15	All	0.099 (0.034)	.006	2,271,787 (10.4)
Maryville	20	30	All	0.170 (0.022)	<.001	579,832 (18.6)
Nixa	20	14	All	0.311 (0.094)	.003	1,147,092 (36.5)
North Kansas City	20	10	All	0.125 (0.046)	.01	640,791 (13.3)

Abbreviations: SE, standard error; NC, not calculated.

^a^ Newey-West estimation technique was used for all the cities with autocorrelation except Columbia and Kansas City to estimate correct standard errors.

^b^ All: seasonal effects, economic condition, and unemployment rate.

^c^ Changes in values were calculated only when the smoke-free ordinance had a significant effect on the revenues of eating and drinking establishments.

## Discussion

Consistent with findings of most peer-reviewed economic studies of smoke-free ordinances, we found that smoke-free ordinances had no negative effect on the local economy. Furthermore, our study showed that smoke-free ordinances were associated with increased revenue in 8 of the 11 cities we assessed. This study provides more evidence to local and state policy makers that the fear of harmful economic effects from passing and implementing smoke-free policies is unfounded.

Implementing effective policy and programs in Missouri to prevent smoking and associated health effects is essential. Smoking caused 9,362 deaths and cost more than $4.8 billion annually in Missouri during 2003-2007 (18). In 2010, the smoking prevalence among adults in Missouri was 21.1%, the 11th highest in the nation. Missouri's tobacco excise tax is 17 cents per pack, the lowest in the nation. In addition, Missouri does not have a statewide smoke-free law.

Enacting a smoke-free ordinance at the local level is a viable approach to prevent smoking and exposure to secondhand smoke. A negative effect on the revenues of eating and drinking establishments has often been cited in opposition to new smoke-free ordinances. This study uses Missouri-specific data from multiple cities to provide empirical evidence to address this concern.

This study had several limitations. First, only combined bar and restaurant taxable sales data were available, and the data were examined in aggregate. It is possible that specific establishments may have lost revenue after enactment of a smoke-free ordinance. However, the main focus of this study was to investigate the effect of ordinances on the local economies. Therefore, the aggregate data used in this study were sufficient. Second, other cost data, such as lost productivity, decreased property value, increased maintenance and replacement of equipment and furnishings, and increased costs of medical treatment of employees, were not included in this study. Previous studies concluded that smoke-free ordinances benefit the restaurant and bar business through greater worker productivity, lower cleaning costs, higher resale value, and potentially lower property and health insurance costs (19,20). Third, the effect of the ordinances on hospitalizations for smoke-related diseases was not included in this study, although the community benefits economically through decreased hospitalizations for myocardial infarction, asthma attacks, stroke, angina, chronic obstructive pulmonary disease, and lung infections (21-23).

Regardless of these limitations, our study used objective taxable sales data and controlled for confounding factors. Therefore, the conclusion of no negative effect is valid, even though we likely underestimated the positive effect of the ordinances due to aforementioned reasons. We also included all Missouri cities that had a smoke-free ordinance and a sufficient number of quarters of taxable sale data in the analysis. The finding of no negative effect was consistent in all 11 cities studied.

The number of local communities that have passed a smoke-free ordinance is growing in Missouri. As of June 2011, 42% of Missouri's population resided in communities that have smoke-free ordinances. The next logical step for public health is to strive for a statewide smoke-free workplace law. The finding of this study provides evidence for garnering support from the general public and legislators by allaying fears that smoke-free ordinances harm business.
