# Erratum, Vol. 9, May 31 Release

**DOI:** 10.5888/pcd9.110277e

**Published:** 2012-07-12

Because of an editing error, we omitted a symbol used for statistical calculations in the article “Economic Effect of Smoke-Free Ordinances on 11 Missouri Cities.” The sentence should have read, “The percentage change between preordinance and postordinance was estimated by exp (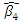) – 1.” This correction was made to our website on June 27, 2012, and appears online at http://www.cdc.gov/pcd/issues/2012/11_0277.htm. We regret any confusion or inconvenience this error may have caused.

